# Does an App a Day Keep the Doctor Away? AI Symptom Checker Applications, Entrenched Bias, and Professional Responsibility

**DOI:** 10.2196/50344

**Published:** 2024-06-05

**Authors:** Ma'n H Zawati, Michael Lang

**Affiliations:** 1 Centre of Genomics and Policy McGill University Montreal, QC Canada

**Keywords:** artificial intelligence, applications, mobile health, mHealth, bias, biases, professional obligations, professional obligation, app, apps, application, symptom checker, symptom checkers, diagnose, diagnosis, self-diagnose, self-diagnosis, ethic, ethics, ethical, regulation, regulations, legal, law, laws, safety, mobile phone

## Abstract

The growing prominence of artificial intelligence (AI) in mobile health (mHealth) has given rise to a distinct subset of apps that provide users with diagnostic information using their inputted health status and symptom information—AI-powered symptom checker apps (AISympCheck). While these apps may potentially increase access to health care, they raise consequential ethical and legal questions. This paper will highlight notable concerns with AI usage in the health care system, further entrenchment of preexisting biases in the health care system and issues with professional accountability. To provide an in-depth analysis of the issues of bias and complications of professional obligations and liability, we focus on 2 mHealth apps as examples—Babylon and Ada. We selected these 2 apps as they were both widely distributed during the COVID-19 pandemic and make prominent claims about their use of AI for the purpose of assessing user symptoms. First, bias entrenchment often originates from the data used to train AI systems, causing the AI to replicate these inequalities through a “garbage in, garbage out” phenomenon. Users of these apps are also unlikely to be demographically representative of the larger population, leading to distorted results. Second, professional accountability poses a substantial challenge given the vast diversity and lack of regulation surrounding the reliability of AISympCheck apps. It is unclear whether these apps should be subject to safety reviews, who is responsible for app-mediated misdiagnosis, and whether these apps ought to be recommended by physicians. With the rapidly increasing number of apps, there remains little guidance available for health professionals. Professional bodies and advocacy organizations have a particularly important role to play in addressing these ethical and legal gaps. Implementing technical safeguards within these apps could mitigate bias, AIs could be trained with primarily neutral data, and apps could be subject to a system of regulation to allow users to make informed decisions. In our view, it is critical that these legal concerns are considered throughout the design and implementation of these potentially disruptive technologies. Entrenched bias and professional responsibility, while operating in different ways, are ultimately exacerbated by the unregulated nature of mHealth.

## Background

Smartphone apps with health-related functions are becoming increasingly popular [1]. A large portion of the global population owns smartphones [2] and many smartphone owners are using widely available apps to monitor their fitness, track their health data, and receive information about illness [3]. Smartphone health apps vary greatly in their functioning and technological complexity. In recent years, a number of highly sophisticated smartphone apps powered by artificial intelligence (AI) have emerged [4]. One prominent subset of AI-powered health apps offers diagnostic information by providing users the capacity to input health status and symptom information [5]. The app, in return, gives users feedback consisting of lists of conditions that might be responsible for the reported symptoms.

Apps of this kind might be capable of increasing access to high-quality medical care, potentially helping to address population inequality in care access. At the same time, symptom checker apps powered by AI also raise serious ethical and legal questions [6]. Chief among them is that AI-powered symptom checker (AISympCheck) apps, to which we will refer in this viewpoint as AISympCheck apps, may work to entrench biases preexisting in the health care system [7] and that they may generate confusion about the legal obligations of health professionals in the course of interacting with these systems [8]. In our view, it is critical that these legal concerns are considered throughout the design and implementation of these potentially disruptive technologies. While it may certainly be the case, AISympCheck apps will, on balance, improve access to preclinical health information in a way that tends to improve health outcomes, this outcome is not assured, particularly if the quality of AISympCheck apps varies wildly. It is important, in other words, to work to ensure that AISympCheck apps are lawfully designed so that their safety and efficacy can be relied upon by users and so that these systems can have beneficial impacts on the broader health care system.

This viewpoint aims to address this idea in 2 parts. First, we provide a brief overview of mobile health (mHealth) and AISympCheck apps by highlighting 2 prominent examples—Babylon and Ada. Second, we outline concerns surrounding (1) the replication and entrenchment of bias and (2) the complication of professional obligations and liability in the use of AISympCheck apps. We conclude by briefly considering how these challenges may be addressed through policy and regulatory reform.

## AISympCheck Apps

### Overview

This first part of the viewpoint provides an overview of AISympCheck apps. We first describe the broader categories of mHealth apps and symptom checker apps of which AI-powered apps are a part. From there, we specify the unique emerging characteristics of symptom checker apps powered by AI. We outline 2 prominent examples as a way of highlighting how these apps function.

### Symptom Checker Apps

Mobile devices are significantly altering the ways individuals interact with health care systems around the world [9]. As we detailed above, smartphone connectivity has become a widespread phenomenon across much of the human population. While there is no universally accepted definition of the smartphone, many commenters track the emergence of the concept to the release of Apple’s iPhone in 2007 [10]. In most of the world’s highly industrialized countries, an excess of 80% of people own a smartphone [11] and across the globe, nearly 4 billion people were smartphone owners in 2020 [12]. These devices are becoming increasingly technologically sophisticated, with features such as high-definition cameras, multiple gigabytes of storage, relatively precise accelerometers, and GPS connectivity quickly becoming standard. These factors combine to make smartphones potentially revolutionary tools for collecting, storing, and sharing health information [13]. Smartphone apps with health-related functions serve a wide variety of purposes, from recording basic fitness data [14] to measuring heart function [15]. Such apps are becoming incredibly numerous, by 1 estimate, more than 350,000 health apps are presently available across Android and iOS systems [16].

Of the several hundred thousand available mHealth apps, approximately 7% of them perform functions broadly resembling clinical diagnosis [17]. These are what we refer to here as “symptom checker applications.” As we outline, this category of mHealth apps, potentially consisting of more than 25,000 examples, is fairly diverse. For our purposes, we follow a conception of the symptom checker category, set out by Lupton and Jutel [18], as a set of apps “by which self-diagnosis can take place.” In most cases, symptom checker apps function in a relatively straightforward way. Users are generally prompted to enter information about symptoms or ailments that are afflicting them. Such information may be processed in a freehand text box or from a drop-down list from which specific symptom descriptions may be selected as appropriate. Symptom information is often supplemented with demographic data such as age, weight, height, or ethnic identity. Following internal app processing, users are usually provided a list of candidate conditions and diseases that may be responsible for the reported symptoms. This information is sometimes supplemented with a probability score expressed as a percentage or some analogous measure indicating how likely a given condition is to be responsible for the specific symptom cluster described by the user. Many symptom checker apps convey a written warning to the effect that information communicated by the app does not constitute a diagnosis and should not be used to replace appropriate clinical care [19]. It is not clear how app users interpret these warnings or whether waivers of nondiagnosis have the meaning in the law that they intend. It may be, for example, that a reviewing court will determine that an app’s tendency to draw causal associations between descriptions of symptoms and disease will constitute a diagnosis in potential contravention of reservations of professional activity to physicians [19].

Symptom checker apps, like mHealth more generally, are a recently emerging and rapidly developing phenomenon in health care. While a comprehensive history of the evolution of symptom checker apps is well beyond the scope of this viewpoint, it may be useful to provide a broad overview of major developments in this space. In 2013, the United Kingdom’s National Health Service published perhaps the most sophisticated early example of a symptom checker app. This app engaged users in a general health assessment, “with users working through a set of questions on health and symptoms, followed by more detailed assessments” [20]. Guidance provided by the app would range from at-home self-care to attending an emergency room [20]. In the period since 2013, symptom checker apps have proliferated. Many of the apps now available are developed and distributed by private entities and businesses, such as the symptom checker app WebMD [18]. This app takes the same basic form as the NHS (National Health Service) Symptom Checker App, though without direct integration into national health care infrastructure. WebMD’s symptom checker interface opens upon download with a disclaimer. Users are warned that while the app provides “useful information,” it is “not a substitute for professional medical advice, diagnosis, or treatment” [21]. User symptoms may be identified according to an animated body plan or by using the app’s database search function [21]. Available symptom descriptions range from cold hands to congestive heart failure. Upon entering symptom descriptions and information about current and past medication use, the app delivers an overview of conditions that might be responsible for the reported symptoms [21]. Potential conditions are presented according to the strength of the relationship between reported symptoms and the identified condition.

### AI-Powered Symptom Checker Apps

#### Overview

This viewpoint draws a further distinction between symptom checker apps and AISympCheck apps. The essential difference in these categories is not elaborate, AISympCheck apps are the subset of symptom checker apps that make use of artificially intelligent processing techniques to deliver information associating symptom inputs with disease. As we outline, there may be important functional differences between those apps holding themselves out in marketing and instructional materials as AI-powered symptom checkers and those actually applying artificially intelligent processing models. There are ongoing debates about what precisely qualifies as an AI app in the mHealth space, debates into which we will not enter here. For our present purposes, we refer to all apps promoted or described by their developers as using artificially intelligent models as AISympCheck apps. Multiple AISympCheck apps have been developed and released in recent years [18] and many have entered popular and academic discussion. By way of example, we provide a short overview of 2 especially prominent AISympCheck apps—Babylon and Ada. We have elected to focus on these as exemplars of the emerging AISympCheck app category, but do not mean to suggest that these are the only presently available AISympCheck apps, nor that Babylon and Ada are more significant than other AISympCheck apps that may be on the market. We mean only for these to serve as examples that illustrate a novel, emerging concept. Babylon and Ada were widely distributed during the COVID-19 pandemic and make prominent claims about their use of AI for the purpose of assessing user symptoms.

#### Babylon

Babylon was first released in the United Kingdom in 2017 [6]. Babylon describes itself as a “revolutionary digital health company,” combining elements of an AI-powered symptom checker and video consult telemedicine [22]. This app deploys an artificially intelligent interface trained on a data set composed of physician clinic notes, user medical records, probabilistic modeling architecture, and a series of diagnostic simulations [23]. Just as in the case of the symptom checker apps described above, a Babylon assessment begins with users searching for symptom descriptions from a standardized list. Upon selecting those most appropriate to a user’s situation, the app interface may pose additional queries. Babylon then provides a list of potentially responsible conditions and advice on the next steps. Such advice might include home care or a physician consult. As Babylon also offers telehealth services, the app may connect users directly with a physician.

Shortly after its release, Babylon claimed its artificially intelligent diagnostic model outperforms human physicians on the Membership of the Royal College of General Practitioners examination, a mandatory standardized test taken by British trainee general practitioners prior to their admission to the practice [24]. But these results have been subject to a certain degree of dispute, with some questioning their validity on methodological grounds [25] and pointing out that Babylon training has been conducted on data primarily collected from young, healthy patients who have been encouraged to register for health services through aggressive direct marketing [26]. With the arrival of the COVID-19 pandemic in early 2020, Babylon’s adoption appears to have expanded. In several Canadian jurisdictions, for example, provincial health systems promoted Babylon use as a way of easing pressure on in-person care. In the provinces of British Columbia, Alberta, and Ontario, telemedicine services on the Babylon app were provided by provincial health insurance schemes [27]. Babylon was not intended in these arrangements to diagnose, treat, or manage COVID-19 directly. Academics and activists raised concerns about Babylon’s promotion by provincial health ministries. Critics note that Babylon’s AI-powered interface has not been independently reviewed for safety and efficacy [28]. Others note that Babylon received initial funding from Saudi Arabia’s sovereign wealth fund, potentially raising conflict of interest concerns for some users [29].

#### Ada

Like Babylon, Ada describes itself as a “world-leading clinical AI” [30]. Launched on the Apple App Store in 2016, Ada reports having conducted more than 20 million health assessments and having more than 13 million active users engaging with the app in 11 product languages [30]. Unsurprisingly, Ada’s user interface bears a great deal of resemblance to Babylon and to the other symptom checker apps described. Ada operates by walking users through a series of prompts to illicit symptom information. The app poses a series of increasingly targeted questions and provides a list of potentially responsible conditions. These are organized probabilistically, with descriptions of the proportion of users who are likely to have a specific condition given the symptoms described. Ada makes fairly sweeping claims about the accuracy of its AI-powered model, a trait as we outlined, it shares with Babylon. Ada’s website, for example, suggests that it “is the most accurate symptom assessment app” [30]. At least 1 study suggests that Ada’s rate of condition suggestion accuracy is consistently just over 70%, as compared with a general practitioner’s accuracy of just over 82% [31]. In the same study, Babylon was determined to have a condition suggestion accuracy rate of about 32% [31].

On the whole, Babylon and Ada perform a similar set of functions in a roughly similar way. There is a level of technical disagreement about how these apps function—whether system modeling can properly be described as AI, how best to measure output accuracy, and how these systems were initially funded and produced. Each of these functional factors raises distinct ethical and legal questions. In the following section, we outline 2 such questions—entrenched bias and professional responsibility.

## Legal and Ethical Considerations: Entrenched Bias and Professional Responsibility

### Overview

In the previous section, we provided an overview of the AISympCheck app. We summarized the functions and conceptual orientation of 2 such apps—Babylon and Ada. In this second part of the viewpoint, we turn to some of the ethical and legal challenges likely to be associated with the proliferation of AISympCheck apps. While an active and growing literature considers the ethical and legal issues raised by the adoption of generalized mHealth apps [32], considerations specific to symptom checker apps have received considerably less attention. Less still has been written on ethical and legal questions that might be particular to the subset of apps powered by AI, though media and activist attention given to certain high-profile AI-powered apps, such as Babylon and Ada, appears to have stimulated a certain level of interest in the field [33]. We focus here on 2 sets of issues that, while challenges for mHealth generally, may have particularly salient implications for AISympCheck apps—entrenched bias and professional responsibility.

### Entrenched Bias

It is well understood that bias, both implicit and explicit, is a persistent and troubling feature of modern health care [34]. Perhaps its most pernicious source is in its relationship to a wide array of social demographic factors, usually called the social determinants of health. These include factors such as race, immigration status, income, and educational attainment, each of which track closely with an individual’s capacity to care, benefit from treatment, and experience complications [35]. Demographic factors also correlate closely with health status and the risk that individuals will become ill in the first place, a reality painfully and unambiguously exposed during the COVID-19 pandemic [36]. Any novel health intervention or technology is likely to raise concerns about its intersection with existing lines of oppression and inequality. Those most vulnerable and most in need of access to improvements in health care delivery are also often those least able to benefit from them.

This is almost certain to be the case in the field of mHealth, in which factors such as income, health literacy, and level of comfort with technology, are likely to have an enormous impact on the capacity of individuals to make use of and benefit from even the most effective and widely available tools [37]. Individuals who are unable to afford a smartphone, for example, are, as per the definition, excluded from the possibility of benefitting from most mobile apps. Likewise, persons with a low level of health or technological literacy might, even where mobile technologies are accessible to them, be unable to effectively use these systems in a beneficial or productive way. Notably, AI systems could be designed in such a way that they identify and correct biased decision-making by reporting to users when a training data set is demographically unrepresentative or otherwise flawed. This solution, though, can only do so much. Reporting that a system relies on unrepresentative training data does not repair the data itself, nor does it answer the question of how much bias is acceptable in the relevant decision-making endeavor. These concerns are surely relevant in the context of AISympCheck apps, though they also take on a secondary and confounding character.

Artificially intelligent systems often face what is sometimes described as a “black box” or inscrutability problem [38]. Much of our most technologically sophisticated AI learns and improves in function over time [39]. It does so by virtue of highly complex, layered programming designed to loosely replicate human neuronal processes [40]. AI systems using machine learning techniques, for example, require minimal human instruction and supervision. These systems, when provided sufficient data, will identify and interpret patterns in a manner similar to human reasoning, though usually significantly more efficiently. Numerous commenters note that this manner of functioning raises serious concerns that AI systems will produce biased decisional outcomes [7]. Bias in this context is likely to originate from the data on which AI systems are trained. Owing to bias preexisting in the health care system, it may be that AI models will work to replicate and entrench inequalities that produce disparate outcomes, rather than remedying them. The essential nature of this problem is often summarized by commenters as the “garbage in, garbage out” phenomenon [41]. Artificially intelligent systems trained on biased data are likely to produce decisional outputs that by necessity reflect that bias.

In the context of AISympCheck apps, this kind of biased output might have a particularly worrying salience. Bias embedded in the health care system owing to compounding social factors are likely also to affect the effective use of modern consumer technologies. We know, for example, that smartphone ownership and rate of use is inconsistent across demographic measures. While 96% of Americans with household incomes above US $75,000 own a smartphone, for example, the rate of ownership among those earning less than US $30,000 in household income is only 76% [42]. Likewise, 93% of Americans with a college degree report owning a smartphone. Only 75% of Americans with a high school diploma or less are smartphone owners [42]. Demographic factors such as level of educational attainment and household income are known to be associated with poorer health outcomes and lower levels of access to the health care system. It is potentially worrying that the increasing adoption of AISympCheck apps may turn out to be poorly suited to addressing such disparities and may, in fact, inadvertently make them worse. Interpreted another way, AI system training that is happening now may turn out to have a significant impact on the future shape of the field. As novel tools and systems are developed and commercially distributed, they may be dominantly affected by presently available (biased) data sets. Current users of AISympCheck apps are not likely to be demographically representative of the population at large, potentially distorting the kinds of results such apps are capable of providing. An AISympCheck app hypothetically trained on data obtained from a data set composed of a disproportionate number of male users, for example, may be inadequately suited to providing diagnostic information addressing conditions primarily affecting nonmale users. This may not be a temporary problem, for even as app use becomes more demographically equitable over time, initial training parameters may have a pernicious and lasting impact.

These considerations raise the possibility that AISympCheck apps will work to entrench and replicate biases preexisting in our health care systems and societies. This is a concern that may limit the capacity of these tools to achieve their ostensible aim of increasing equitable access to health information and care.

### Professional Responsibility

Another factor keeping AISympCheck apps from realizing their anticipated clinical potential is the possibility they will integrate poorly into the professional practice of medicine. AISympCheck apps raise a cluster of questions surrounding professional and regulatory responsibility. It is unclear, for example, whether such apps are subject to the same safety and efficacy review usually carried out prior to the regulatory approval of novel medical devices. It is likewise unclear whether physicians ought to recommend AISympCheck apps to their patients. Where prescribing a mobile app could be a clinical benefit, it is unclear how a physician ought to select an appropriate system from the many thousands on the market. Patients, moreover, are likely to use AISympCheck apps outside of their relationships with doctors. They may, from time to time, present app-generated information or diagnoses in the clinic. It is not obvious how a physician ought to conceive of or manage such information. Physicians are generally concerned about the increasing prevalence of mHealth apps in the health care system. In one study, two-thirds of Australian general practitioners had used mHealth apps in some capacity in their practice and more than half had recommended apps to their patients [16]. But their use was far from universal. Physicians might be hesitant to use and recommend mHealth apps for a variety of reasons. Byambasuren et al [16] find 2 major reasons for avoiding the clinical adoption of mHealth apps. First, an absence of adequate knowledge about which apps are most effective (n=372, 60%) and second, the lack of trustworthy sources through which to access health apps (n=96, 15%). Surveyed physicians indicated that increased mHealth training (n=243, 30%) and lists of apps evaluated for safety and efficacy by a reliable source (n=224, 28%) would increase comfort in the clinical adoption of mHealth apps [16]. These findings suggest that many physicians are (perhaps sensibly) fearful of inadvertently adopting ineffective, potentially harmful apps in their practice.

Without proper training and guidelines to follow, it is quite unreasonable that physicians could be expected to determine for themselves which mHealth apps are best suited to serving a patient’s interests and how these apps ought to be used in the provision of care. There are simply too many apps with too many features and too many potential complications. Even in highly specific, discrete subspecialties, the number of available apps is rapidly increasing. Against this backdrop, formal law provides little in the way of guidance for clinicians. The mHealth apps, as we suggested, are typically not governed by existing formal regulations. Symptom checker apps in Canada, for example, are explicitly excluded from regulatory oversight [43]. Similar situations can be found in jurisdictions around the world [44]. There are a range of reasons that might explain the relative paucity of regulation surrounding symptom checker apps, including that such apps are exceptionally technically versatile and may be developed in a diverse number of settings, from hospitals to corporations to the homes of individuals [44]. Just as the number and diversity of apps create difficulty for clinicians in deciding whether to use apps in their medical practice, so too might it generate challenges for regulators.

In the absence of formal regulation, advocacy organizations and professional bodies have taken up some of the work of controlling the use of mHealth apps through professional responsibility regimes, by which we mean the quasi-judicial procedures according to which physicians and other health care practitioners are regulated by the professional bodies of which they are members. The United Kingdom’s National Health Service, for example, maintains a searchable database of apps reviewed for clinical safety, accessibility, usability, and technical stability [45]. The App Library serves primarily as an advisory resource for clinicians and the public. It does not control clinician activities, does not provide guidance on appropriate clinical applications, and does not purport to serve as an exhaustive list of trustworthy mHealth resources. Taking another approach, the Canadian Medical Association (CMA) in 2015 released its “Guiding Principles for Physicians Recommending Mobile Health Applications to Patients” [46,47]. Among other things, this CMA policy notes that mobile apps may permissibly be used to complement, but should never replace physician care. It further recommends that physicians “prescribe” apps to patients with the primary objective of enhancing the safety or effectiveness of patient care [47]. Notably, neither the NHS App Library nor the CMA policy outlines specific rules for symptom checker apps. In the case of the NHS App Library, reviewed apps are organized into 16 categories, none of which refer precisely to the symptom checker designation [45]. Using the App Library search function and the fixed term “symptom checker” returns only 1 result, My Possible Self: The Mental Health App, a self-help app for managing fear, anxiety, and stress [45].

While the nonexistence of regulation and guidance for the clinical use of mHealth apps is a generally pervasive problem [44], it may be that its effects are especially pronounced in the context of apps powered by AI. There are at least 2 reasons for this. First, AISympCheck apps might be significantly more powerful than existing consumer health technologies. Apps using artificially intelligent processing techniques, for example, may be able to deliver diagnostic information with unprecedented levels of accuracy and specificity relative to other mass-market digital tools. This may promote high levels of public adoption while also increasing the clinical use of these systems. But even potentially accurate systems will likely sometimes produce errors, an eventuality that could be heightened by the absence of regulation of AISympCheck apps. The possibility that symptom checker apps will provide inaccurate information poses a clear potential risk to users. App-mediated misdiagnosis might also cause confusion in medical practice. Care providers engaged in patient triage, for example, might have a difficult time accurately assessing patients presenting in the clinic with erroneous diagnostic information. Second, many AISympCheck apps are likely to be inscrutable in the sense, described above, that programmers and reviewers will be unable to understand how these systems technically operate. Physicians using or interacting with inscrutable decision systems may be unable to satisfactorily explain diagnostic outputs to their patients. Technical inscrutability may likewise impede physicians and regulators from systematically differentiating between high-quality, reliable apps and low-quality, unreliable apps. Inscrutability can also be expected to raise difficult questions about who, ultimately, is responsible should AI-powered tools cause injury. Complex and intersecting systems of medical malpractice, professional responsibility, and manufacturer liability may be ready to address injury caused by AISympCheck apps.

### Addressing Entrenched Bias and Professional Responsibility

We contend that AISympCheck apps may work to entrench existing bias and greatly complicate the conventional approach to professional responsibility in the context of novel health technologies. These and related lines of concern are likely to attract the attention of scholars, policy makers, and activists. To be sure, these are far from the only legal and ethical concerns raised by the increasing use of AISympCheck apps. These tools also raise important questions about privacy, data processing, commercialization, premarket review, and the privatization of health care. Indeed, several authors have highlighted these issues, outlining challenges associated with an absence of clarity surrounding data custodianship when users interact with AISympCheck apps [27]. All of this is made more confounding by the tendency of AISympCheck apps to enter the market absent any prior, independent regulatory review. As we outlined above, these systems are effectively unregulated in Canada and elsewhere.

Entrenched bias and professional responsibility are distinct kinds of problems. Though they operate in different ways, both are exacerbated by the unregulated nature of mHealth. There are no clear rules about whether or how AISympCheck apps should be used. This confounds and complicates the question of professional responsibility in a fairly direct way, but so too does it pose additional problems for entrenched bias. As we outlined above, the problem of entrenched bias in AISympCheck decision-making is in large part a problem associated with the quality of data on which these systems are trained. Conceived as a challenge that operates as a function of the characteristics of training data, 3 general kinds of solutions might be helpful.

First, technical safeguards embedded into AISympCheck app programming could operate to mitigate or correct biased outcomes. But technical safeguards alone might be an insufficient remedy, for programmers will need to make assessments about what constitutes bias in the relevant circumstances. While not a problem in itself, often the most pernicious forms of discrimination may be those not initially recognized. Bias may operate on a complex web of interrelated factors; it might not be immediately obvious, for example, that an apparently innocuous decision has a discriminatory effect. These forms of bias could be overlooked in a merely programmatic response.

Second, AISympCheck systems could be trained principally on unbiased or neutral data. In response to the garbage in, garbage out phenomenon, it may be tempting to stress that we simply should not feed garbage into the system. Of course, this would massively oversimplify the entrenched bias challenge, while also again prompting programmers to evaluate what does or does not constitute a biased data set. This does not solve so much as punt the problem. One reaction to the garbage in, garbage out phenomenon could be that it is not a problem unique to AI systems but is a prevailing concern across medicine. We agree but would stress that the unexplainable character of many of our best AI tools makes the phenomenon potentially more pernicious than in other settings. If we do not know how a system processes data to arrive at a conclusion, it may be especially difficult to measure when the data in issue is “garbage.”

Third, AISympCheck apps could operate according to a system of regulation and guidance that empowers users to make informed decisions about which apps are safe and reliable, that delineates the responsibilities of clinicians and others using AISympCheck apps in their practice, and that reviews individual apps periodically to ensure their proper function.

While this line of solution does not directly address the core set of issues in biased AI-mediated decision-making, it may be the best medium-term option for reducing discriminatory outcomes in the AISympCheck app context. It has the additional advantage of also working to address the second kind of challenge we identified above, namely that there persists a great deal of uncertainty about professional responsibility for the use of AISympCheck systems. In addressing both challenges, the most urgent unmet need is the absence of regulation or guidance specifically addressing AISympCheck apps. Questions about both bias and responsibility can be expected to fester in the absence of a framework that permits app users to evaluate and make decisions about how AISympCheck apps ought to be used. A reasonable place to begin, particularly in the Canadian context, is for professional medical associations to develop guidance for their members on the use of novel mHealth apps, especially those of the AISympCheck variety. Professional medical associations have played a long and essential role in working to structure the practice of medicine, guide clinicians in the performance of their obligations, and protect patients from risk [46]. In the Canadian context, provincial medical colleges that oversee the professional certification and clinical practice of physicians may have a unique role to play in guiding the use of AISympCheck apps. Medical professionals are, after all, primary reservoirs of expertise on the clinical use of novel health technologies and on the ways such technologies might affect physician-patient relationships. We, therefore, suggest that the dual challenges of entrenched bias and professional responsibility could begin to be addressed through medical college guidance that minimally (1) outlines factors that would assist in determining which apps are safe, reliable, and as best as possible avoid discriminatory outcomes; (2) provides guidance for physicians on whether and how to recommend AISympCheck apps to their patients; and (3) considers how physicians should manage information provided by an AISympCheck app when a patient presents with a mobile-mediated diagnosis.

## Conclusions

This viewpoint introduces the concept of the AISympCheck app, an mHealth app powered by AI and designed to provide users with information on a broadly diagnostic character. We outline 2 legal and policy challenges to which AISympCheck apps are likely to be especially susceptible—entrenched bias and professional responsibility. These issues underscore the critical importance of addressing the regulatory and guidance lacuna existing in this space, as well as the necessity of continued research that monitors the quality, safety, and efficacy of AISympCheck systems. We suggest in this viewpoint that AISympCheck apps will likely have increasing clinical implications and that medical colleges could play a central role in developing guardrails for their use. These guardrails will not only work to provide clarity to physicians and health systems using AISympCheck apps in clinical practice but to the app developers as well. To be sure, the regulation of clinical AI cannot by itself remedy all of the challenges described in his viewpoint. Problems surrounding biased training, for example, have deep structural and sociological roots that likely cannot be solely addressed in AI regulation. We nevertheless propose that forward-looking regulation and guidance in this space will attenuate many of the more serious risks, will help to facilitate the lawful design of these potentially disruptive tools, and ensure that their use serves the interests of patients and the public.

## Abbreviations

AI: artificial intelligence
AISympCheck: AI-powered symptom checker
CMA: Canadian Medical Association
mHealth: mobile health
NHS: National Health Service

## Data Availability

Data sharing is not applicable to this paper as no data sets were generated or analyzed during this study.
